# Paleozoic–Mesozoic Eustatic Changes and Mass Extinctions: New Insights from Event Interpretation

**DOI:** 10.3390/life10110281

**Published:** 2020-11-14

**Authors:** Dmitry A. Ruban

**Affiliations:** K.G. Razumovsky Moscow State University of Technologies and Management (the First Cossack University), Zemlyanoy Val Street 73, 109004 Moscow, Russia; ruban-d@mail.ru

**Keywords:** biotic crisis, global sea level, life evolution

## Abstract

Recent eustatic reconstructions allow for reconsidering the relationships between the fifteen Paleozoic–Mesozoic mass extinctions (mid-Cambrian, end-Ordovician, Llandovery/Wenlock, Late Devonian, Devonian/Carboniferous, mid-Carboniferous, end-Guadalupian, end-Permian, two mid-Triassic, end-Triassic, Early Jurassic, Jurassic/Cretaceous, Late Cretaceous, and end-Cretaceous extinctions) and global sea-level changes. The relationships between eustatic rises/falls and period-long eustatic trends are examined. Many eustatic events at the mass extinction intervals were not anomalous. Nonetheless, the majority of the considered mass extinctions coincided with either interruptions or changes in the ongoing eustatic trends. It cannot be excluded that such interruptions and changes could have facilitated or even triggered biodiversity losses in the marine realm.

## 1. Introduction

During the whole Phanerozoic, mass extinctions stressed the marine biota many times. They triggered disappearances of numerous species, genera, families, and even high-order groups of marine organisms, and they were often associated with outstanding environmental catastrophes such as global events of anoxia and euxinia, unusual warming and planetary-scale glaciations, massive volcanism, and extraterrestrial impacts. Mass extinctions were highly complex events, with the intensity amplified by the interplay among atmosphere, oceans, geologic activity, and extraterrestrial influences. The relevant knowledge is huge, and it continues to grow. Importantly, this knowledge growth is indivisible from the progress in the understanding of marine (paleo)ecosystems and (paleo)environments, both at the global and local scales. The most important contributions that formed the very fundamentals of what can be called “mass extinction science” were made by Bambach [1], Benton [2,3], Clapham and Renne [4], Elewa and Abdelhady [5], Erwin [6], Hallam [7], Jablonski [8], Holland [9], Melott and Bambach [10], Racki [11], Rampino and Caldeira [12], Raup and Sepkoski [13], Thomas [14], Twitchett [15], and Wignall [16]. Hundreds of other researchers have also contributed substantially and focused on particular catastrophic events, fossil groups, or extinction factors.

Eustatic (global sea-level) changes have been regarded as a debatable cause of mass extinctions for decades [17,18,19,20,21]. However, currently, there is not consensus about the relevance of this cause. This is due to stratigraphical record biases, the complexity of mechanisms linking eustasy and marine biodiversity changes, and “chaos” in global sea-level interpretations [22]. Often, restriction of the analyses to only regional or even local records and confusion between transgressions–regressions (“horizontal” changes) and sea-level rises–falls (“vertical” changes) also matter. Moreover, substantial improvements in the geologic time scale and eustatic reconstructions require permanent re-consideration of earlier research, which is undermined by the unpopularity of theorizing in modern geology [23]. One may even question the very sensibility of special studies of the eustatic factor of mass extinction. Nonetheless, this factor cannot be ignored. Whether global sea-level changes were able to trigger mass extinctions, it appears undisputable that the position and tendencies of the global sea level was one of the environmental conditions that could either reduce or facilitate the biodiversity loss during mass extinction events. In other words, the eustatic setting of mass extinctions requires proper examination. A significant question is whether this research direction should shift from qualitative interpretations (visual interpretations of curves depicting changes of biodiversity and sea level) to more quantitative (statistical) analyses. Although the latter seem to be more advanced than the former, three reasons should be taken into consideration. First, statistical analyses need conceptual frameworks. Second, the selection of biotic crises for analysis is a subjective procedure due to various biases in the available knowledge. Third, qualitative analyses of the mass extinction causes persist in current geoscience research, whereas quantitative analyses are essentially not so new [24,25]. The importance of the periodicity/cyclicity studies by Melott and Bambach [10], Rampino and Caldeira [12], Roberts and Mannion [20], Boulila et al. [26], Gillman and Erenler [27], Lipowski [28], McKinney [29], Rampino [30], and Tiwari and Rao [31] is undisputable. However, the place for qualitative research still exists, especially when new interpretative approaches are employed.

Although it is more or less clear when the Phanerozoic mass extinctions took place, it is always a challenge to choose eustatic reconstruction for reference. Many studies have focused on regions with “ideal” (much complete and detailed) stratigraphic and sedimentary records of mass extinction intervals for deciphering the paleoenvironmental factors. In these cases, they depended on regional sea-level reconstructions. However, it is myopic to ignore well-established, global-scale eustatic reconstructions, especially in light of significant advancements in their development during the past decade. Particularly, Haq [32,33,34] and Haq and Schutter [35] updated and justified from a stratigraphical viewpoint their previous reconstructions of the Paleozoic–Mesozoic global sea-level changes [36,37]. Their new reconstructions comprise a precious source that seems to be essential for understanding the importance of the eustatic factor in mass extinctions. Such knowledge should be considered very seriously and not put aside due to the “fact-only”, “over-empirical” ethos of contemporary geological research [23]. The scope of the present paper is to undertake a reconsideration of the relationships between the fifteen Paleozoic–Mesozoic mass extinctions (including all the so-called “Big Five”) with the forementioned new eustatic reconstructions. It appears urgent to shift from simplistic comparisons between the global sea-level rises/falls and the major episodes of biodiversity losses towards more advanced analytical approaches like an episodic event analysis [38]. This allows for a new, broader vision of these relationships. This interpretation is based on numerical estimates of the past sea-level changes and employs a graphical analysis of trends based on these estimates. The purpose of this paper is not to provide an interpretation of the mechanisms beneath mass extinctions, but rather to provide a general framework for their relationships with sea-level changes.

## 2. Methodology

**Figure 1:**
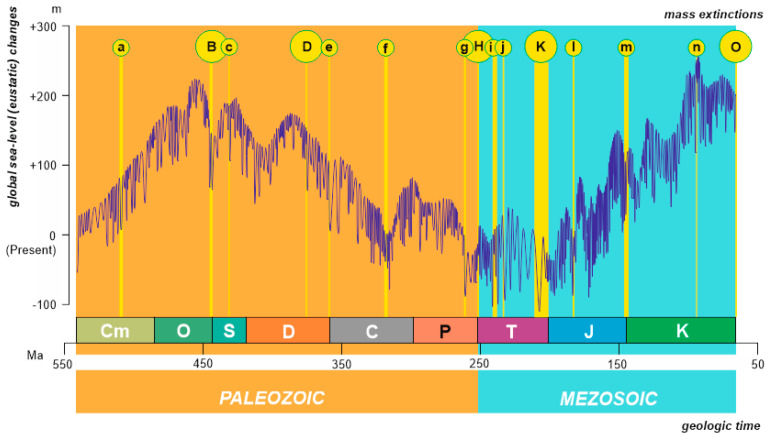
Eustatic changes (deep blue line) and mass extinctions (yellow stripes) during the Paleozoic–Mesozoic (see the main text for data sources). The letters identifying the mass extinctions are according to Table 1. A high-resolution version of this figure with indication of eustatic trends is available as Supplement 1.

For the purposes of the present study, the newest version of the Paleozoic–Mesozoic eustatic reconstruction is taken into consideration. First, the four curves reflecting the Paleozoic, Triassic, Jurassic, and Cretaceous global sea-level changes [32,33,34,35] are here combined into a single curve and justified to the same scale as eustatic changes. This combination is a simple procedure of curve digitizing, joining, and re-scaling. Special attention was paid to junction points at the boundaries of the Paleozoic era and the three Mesozoic periods: this was greatly facilitated by the fact that the reconstructions in the above works are not strictly limited to the stratigraphical boundaries of the considered intervals of geologic time, but are shown along with the terminations of the adjoining curve (e.g., the Triassic curve is shown together with the end of the Paleozoic curve and the beginning of the Jurassic curve). Then, the joint curve was justified to the newest version of the geologic time scale adopted and continually improved by the International Commission on Stratigraphy [39]. As “short-term” eustatic changes are considered, the resulting curve looks rather detailed (Figure 1). Although several curves [32,33,34] refer to periods and one curve [35] refers to an era, these can be combined because all four curves are justified to the geochronological time scale, i.e., they have essentially the same scaling and resolution.

A total of 15 mass extinctions known from the Paleozoic–Mesozoic marine fossil record were plotted against the compound eustatic curve (Table 1). These include all the “Big Five” mass extinctions, and some relatively less impactful events. The magnitude of these events is not discussed herein, as it remains debatable which mass extinctions should be judged as major, and which are instead minor (e.g., see [1,5]). Of course, it cannot be excluded that some biotic crises are still missed by geologists. Nonetheless, the present study deals with a representative set of mass extinctions for evaluating their relationships with eustatic changes. To justify the position of the chosen mass extinctions on the eustatic curve, the most recent views on the age of the mass extinctions (either numerical age or stratigraphical interval, or both) were taken into account (Table 1). The initial works by Haq [32,33,34] and Haq and Schutter [35] allowed for the correspondence between the global sea-level cycles and both geochronological and chronostratiographical scales, thus facilitating a precise positioning of the mass extinction intervals relative to both scales (Figure 1).

**Figure 2:**
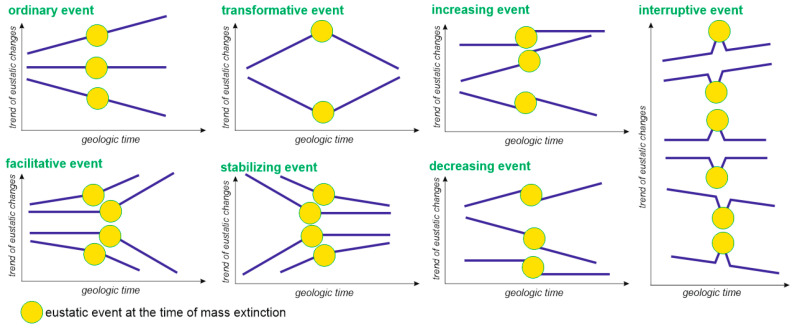
Classes of episodic events (yellow circles) that mark changes of eustatic trends (deep blue lines) (modified from [38]); several events are shown on the same graphs to illustrate the variety of situations.

Analytical procedures of this study follow the principles of the analysis of episodic events [38]. Eustatic changes are typical events of this kind. Each particular event can be characterized in several dimensions. First of all, it can be recognized generally as a global sea-level rise or fall. The relevance of mass extinctions to the general view of eustatic events was discussed earlier by Hallam and Wignall [17], and their conclusions need to be updated in light of the new reconstructions. However, “deeper” interpretations are possible when eustatic events that marked mass extinction intervals are compared to the trends of such changes. On the one hand, episodic events are either regular (their magnitude is more or less similar and they do not deviate much from the trend) or anomalous (exhibiting an outstanding magnitude and/or significant deviation from the general trend). On the other hand, episodic events may coincide with the trends in different ways, and these may be ordinary, facilitative, transformative, stabilizing, increasing, decreasing, and interruptive events (Figure 2). Importantly, mass extinctions are also episodic events [38], which are always negative (biodiversity drops) and anomalous (very high extinction rate and outstanding biodiversity drops). Although some (if not all) mass extinctions were short-term events, and their duration was less than that of the considered eustatic events, this is not too big a limitation because this study emphasizes the correspondence of mass extinctions to eustatic tendencies, not particular, short-term fluctuations.

Each eustatic event that happened within the occurrence of a given mass extinction is characterized generally (i.e., as a global sea-level rise or fall, lowstand or highstand) and in relation to eustatic trends and their changes (regular or anomalous, ordinary or transformative, facilitative or stabilizing). Haq [32,33,34] and Haq and Schutter [35] outlined that their eustatic reconstructions allowed for the recognition of (at least) two orders of global sea-level changes that were provisionally labeled as “short-term” and “long-term” changes. These orders are well visible in the joint curve employed for the purposes of the present study (Figure 1). If so, it seems reasonable to also conduct the present analysis of episodic events on two levels. The “short-term” record is limited to geologic epochs and periods, and the “long-term” record corresponds to several geologic periods or eras. For instance, the “short-term” trend is the global sea-level fall across the Silurian/Devonian transition, and the “long-term” trend is the global sea-level rise during the Jurassic–Cretaceous. Biotic crises were geologically very short events, but it cannot be excluded that they occurred due to biosphere vulnerability triggered by some long-term processes.

## 3. Results

From several biotic perturbations of the Cambrian, the end-Series 2 crisis deserves special attention because of its strength [40,66,67]. This event corresponds to a pronounced eustatic fall followed by a big rise, as depicted by Haq and Schutter [35]. However, this event was not anomalous, as similarly strong fluctuations took place earlier and later during the Cambrian (Figure S1). The second half of this period was characterized by the well-established “short-term” and “long-term” trends of the global sea-level rise, and the event did not challenge these trends (Figure S1). Therefore, it was an ordinary event.

A really severe mass extinction marked the end of the Ordovician, and it is known as the Hirnantian event [41,42,68,69,70]. This biotic catastrophe corresponds to an outstanding eustatic fall that was followed by a significant rise; the other fall of smaller magnitude, but reaching an even lower negative peak, took place soon after the first fall [35]. Undoubtedly, both these falls were anomalous relative to the preceding and subsequent events (Figure S1). In the “short-term” record, the end-Ordovician eustatic event was evidently transformative, as it marked the change of the trend (Figure S1). However, this event was interruptive in the “long-term” record because it broke the general trend of the global sea-level fall that was established in the mid-Ordovician and ended in the Permian, but this trend was restored soon after the end-Ordovician catastrophe (Figure S1).

A mass extinction took place at the Llandovery/Wenlock transition in the first half of the Silurian, and it is known as the Ireviken event [43,71,72,73,74]. This event corresponds to a significant eustatic fall that seems to be unusual for the Silurian, as depicted by Haq and Schutter [35]. Consequently, this event was anomalous (Supplement 1). In the “short-term” record, this eustatic event was decreasing (Supplement 1). Its position relative to the “long-term” trend is less clear because this eustatic fall overlapped with a longer, although relatively weak, eustatic lowstand already established in the Ordovician (Supplement 1). This lowstand was evidently interruptive, but the role of the smaller lowstand at the Llandovery/Wenlock transition is unclear, and it is provisionally defined as quasi-interruptive.

A significant mass extinction occurred at the Frasnian/Famennian transition in the Late Devonian, and it is known as the Kellwasser event [44,75,76,77,78]. According to the reconstruction by Haq and Schutter [35], this biotic catastrophe corresponds to relatively low-magnitude fluctuations of the global sea level followed by a pronounced fall. This fall was anomalous in comparison to the other Devonian eustatic changes, although similarly strong falls and rises became typical already in the second half of the Late Devonian (Supplement 1). Notably, this event does not coincide with eustatic trend change in the “short-term” and the “long-term” records (Supplement 1), and, thus, it looks like an ordinary event. However, anomalous events cannot be ordinary by definition [38], and as such it is more correct to classify this event as interruptive.

Another mid-Paleozoic mass extinction occurred at the Devonian/Carboniferous transition, and it is known as the Hangenberg event [44,79,80,81,82]. This biotic catastrophe corresponds to a rather significant global sea-level fall shown by Haq and Schutter [35]. The magnitude of this fall was unprecedented for the Devonian, but not so uncommon for the following Mississippian Epoch (Supplement 1). Therefore, the event can be classified as only relatively anomalous. In the “short-term” and “long-term” records, it did not produce any principal change of the trends because the global sea level continued to fall (Figure S1). This event is interruptive due to its relatively anomalous character.

There was a mass extinction near the end of the Mississippian, and this occurred in the late Serpukhovian [45,46,83,84,85]. It corresponds to moderate eustatic fluctuations and preceded the outstanding mid-Carboniferous eustatic minimum [35]. The noted fluctuations were typical for the late Mississippian (Figure S1), and, thus, the event was regular. In the “short-term” record, this mass extinction occurred when the global sea level tended to fall, but the intensity of this fall diminished slightly (before the outstanding minimum that occurred later). Therefore, the eustatic event was stabilizing. In the “long-term” record, this mass extinction coincided with a lengthy lowstand, which, however, broke the general tendency of the global sea-level fall through the Middle–Late Paleozoic, and as such the eustatic event is interruptive.

A mass extinction that was a “prelude” to the end-Permian catastrophe occurred at the end of the Guadalupian Epoch (Capitanian Stage) [47,48,86,87,88]. The timing of the event remains unclear, and it is not excluded that it occurred earlier, i.e., in the mid-Capitanian [89,90]. In this study, the very end-Guadalupian position of the catastrophe is considered, but this does not mean that the forementioned alternative position is rejected. This mass extinction coincided with a significant eustatic fall when the Paleozoic eustatic minimum was reached [35]. Undoubtedly, this event was anomalous (Figure S1). In the “short-term” record, this fall was a stabilizing event, which marked the change from the trend of the global sea-level fall from the mid-Permian to the relative stability during most of the Lopingian, except at the very end (Figure S1). It is highly challenging to define this event in the “long-term” record because the trends at the Paleozoic–Mesozoic transition are uncertain. Despite the evident eustatic rises in the Early and Middle Triassic, the global sea level did not reveal a strong tendency to rise until the beginning of the Jurassic (Figure S1). Therefore, it can be hypothesized that the Triassic overall was a “long-term” lowstand established near the end of the Guadalupian. If so, the end-Guadalupian eustatic fall was also a stabilizing event in the “long-term” record.

The most severe mass extinction stressed and almost “erased” the world marine biota at the Permian/Triassic boundary [50,51,91,92,93,94,95]. This catastrophe corresponds to relatively weak eustatic fluctuations as depicted by Haq and Schutter [35] and Haq [34]. There was a moderate global sea-level rise followed by a similarly moderate fall; the magnitude of these changes was significantly smaller than that of the preceding and subsequent fluctuations (Figure S1). Consequently, the event was far from being anomalous. The larger global sea-level rises and falls that occurred just before and just after the event were also regular. In the “short-term” record, the end-Permian mass extinction coincided with the eustatic changes that marked an acceleration of the general trend of the global sea-level rise (Figure S1). Consequently, a facilitative event can be postulated. In the “long-term” record, the end-Permian mass extinction occurred soon after the start of the Lopingian–earliest Jurassic lowstand and, therefore, the eustatic changes coinciding with this catastrophe can be classified as ordinary, as they did not distort any trend.

In the Ladinian Stage of the Middle Triassic, the occurrence of a new mass extinction has been argued recently [5,52]. Although this biotic perturbation still needs to be studied in detail, its catastrophic nature has nonetheless been revealed. According to Haq [34], this mass extinction corresponds to several eustatic fluctuations, from which the only global sea-level rise in the very beginning was really significant. All these changes are rather typical for the Middle Triassic (Figure S1), and, thus, these can be classified as regular. In both the “short-term” and “long-term” records, these eustatic events did not challenge the trends (Figure S1) and are, therefore, ordinary.

The other extinction event occurred in the Carnian Stage of the Late Triassic [53]. This biotic perturbation was reported very recently, but the arguments in favor of its existence are really strong. According to Haq [34], this event corresponds to a strong eustatic fall. However, fluctuations of the comparable magnitude were typical for the Late Triassic (Figure S1), and, thus, the noted fall is not anomalous. Similarly to the previous biotic crisis, this fall did not change or disrupt “short-term” and “long-term” trends (Figure S1), i.e., it was an ordinary event.

The next big mass extinction is known from the end-Triassic, and it occurred during most of the Rhaetian Stage [54,55,96,97,98,99,100]. This biotic catastrophe corresponds to an outstanding global sea-level fall followed by a similar rise and subsequent changes of lesser magnitude as depicted by Haq [33,34] (Figure S1). Undoubtedly, the above fall and rise constitute an anomalous event. In the “short-term” record, this event superimposed with the general tendency of the global sea-level fall that was established in the beginning of the Late Triassic and culminated in the Early Jurassic (Figure S1), i.e., this was an interruptive event. In the “long-term” record, this event occurred before the end of the Lopingian–earliest Jurassic lowstand (Figure S1), i.e., it was interruptive relative to the general trend.

The Early Jurassic was marked by a mass extinction at the very beginning of the Toarcian Stage [57,58,59,60,101,102,103,104]. This catastrophe corresponds to a significant global sea-level rise that was followed by an even stronger fall [33]. This eustatic event is somewhat anomalous, although the magnitude of the Early–Middle Jurassic eustatic changes was relatively big (Figure S1). In the “short-term” record, this event is marked by a trend change (Figure S1), and, thus, the event can be classified as transformative. In the “long-term” record, this mass extinction coincided with a pronounced lowstand superimposed on the Jurassic–Cretaceous trend of the global eustatic rise; after this lowstand, the trend restarted from the lower point (Figure S1). This means this eustatic event was decreasing.

The Jurassic/Cretaceous transition was marked by a mass extinction that, surprisingly, has been studied significantly less than many other Mesozoic biotic perturbations [60,61]. This scant attention can be explained partly by the still problematic stratigraphy of this transition interval (some improvements have been made very recently [105,106,107]); a sensible and well-argued suggestion (although requiring broad discussion before final approval) of the replacement of the period boundary has been made recently [108], but even this solution will not allow us to correlate better the biodiversity losses and turnovers at the planetary scale. According to the reconstruction by Haq [32,33], this mass extinction corresponds to several eustatic fluctuations, the magnitude of which was comparable to that of many other eustatic events of the latest Jurassic–earliest Cretaceous (Figure S1). Consequently, the noted fluctuations were not anomalous. Taken together, the global sea-level changes at the mass extinction interval constitute a particular composite event, i.e., a weak eustatic fall. This was also not an anomalous event. In the “short-term” record, this composite event superimposed with the trend of global sea-level fall established in the Late Jurassic and lasted until the mid-Early Cretaceous (Figure S1). If so, it was an interruptive event. In the “long-term” record, this event marked the beginning of a lengthy break of the Jurassic–Cretaceous trend of the global eustatic rise (Figure S1), i.e., this event was interruptive.

In the Late Cretaceous, a mass extinction occurred near the end of the Cenomanian Stage, which is known as the Bonarelli event [62,63,103,109]. This biotic perturbation corresponds to small-scale eustatic fluctuations that followed a very strong global sea-level rise [32]. The noted fluctuations were very regular, and even the noted rise is difficult to classify as anomalous because fluctuations of the same magnitude were typical for the entire first half of the Late Cretaceous (Figure S1). In the “short-term” record, the eustatic rise at the onset of the mass extinction interval marked the change from a trend of global sea-level fall to that of a rise (Figure S1), i.e., these events were transformative. In the “long-term” record, attention should be paid to the small-magnitude fluctuations that occurred just before the Cretaceous eustatic maximum, at which the Jurassic–Cretaceous trend of the eustatic rise ended (Figure S1). With respect to this observation, the sea-level changes linked to the late Cenomanian biotic catastrophe are transformative.

Finally, a severe and definitely the best-known mass extinction occurred in the very end of the Cretaceous [65,110,111,112,113,114,115]. According to Haq [32], this catastrophe corresponds to a minor global sea-level fall, which was a very regular event (Figure S1). In both the “short-term” and “long-term” records, the event did not distort any trend, and as such it was ordinary. Even when expanding the mass extinction interval so as to include the moderate-scale (relative to the other Late Cretaceous events) fluctuation before the Cretaceous/Paleogene boundary (significant fall and significant rise—see Supplement 1), this fluctuation is neither anomalous nor interruptive.

## 4. Discussion

Taking into account the most recent eustatic reconstructions [32,33,34,35] allows for realizing different relationships between the Paleozoic–Mesozoic mass extinctions and the global sea-level changes (Table 2). Although a falling sea level was found in more than half of the cases, the different eustatic settings were not uncommon. Moreover, it is also evident that many biotic catastrophes occurred when the eustatic changes were weak. Generally, no definitive relation can be found, and two inferences are possible. First, even if eustatic changes were able to trigger mass extinctions, a eustatic cause can be established only by a limited number of cases. In other words, this cause cannot represent a universal explanation for all the mass extinction events. This interpretation echoes an earlier conclusion by Hallam and Wignall [17] (Table 2). Therefore, improvements in both the eustatic reconstructions and the geologic time scale during the past 20 years did not make the eustatic cause of biodiversity losses more plausible. Second, the global sea level and its changes determined environmental conditions in which mass extinctions took place.

The pioneering analysis of episodic events of eustatic changes provides somewhat ambiguous evidence. On the one hand, many events were not anomalous (Table 3), i.e., the most relevant mass extinctions occurred when the global sea-level did not experience extraordinary changes. This represents strong evidence against the eustatic cause of many, if not all, mass extinctions. On the other hand, the majority of the mass extinctions were linked to eustatic events that either interrupted or changed the “short-term” and/or “long-term” trends of the global sea-level variations (Table 3). This evidence seems to be enough to state that biotic catastrophes tended to associate with eustatic events that are not ordinary. The results of the episodic event analysis do not permit us to neglect the potential importance of eustatic changes to the mechanisms of mass extinctions. In light of the new findings, it is logical to hypothesize that rather than global sea-level rises/falls, interruptions and changes of eustatic trends could indeed facilitate or even trigger many Paleozoic–Mesozoic mass extinctions. Although the full-argued explanation of the relevant mechanisms is yet to be developed, one can speculate that interruptions of epoch-long and era-long tendencies of the global sea-level changes might be able to stress marine communities via “putting” the latter into really new conditions that did not exist for a long time before. Interrupted and changed trends created such new conditions. In order to illustrate this idea, a simple, conceptual example can be given as follows. Marine biota evolved over a long time in conditions of fluctuating, but generally rising, sea level, and its development was framed by adaptation to the opening of new niches and increasing connectivity of water masses. If a trend of sea-level fall was established later, this means marine biota needs adaptation to niche closure and fragmentation of water masses. Indeed, these adaptations are developed, but this requires changes in the ecosystem organization or distribution, which makes this biota vulnerable to negative external influences and intrinsic factors during the transition from the past to modern conditions. Anyway, this paper is not aimed at interpretations of the mechanisms beneath mass extinctions, although the proposed framework for their relationships with sea-level changes might prove useful for further interpretations of this issue.

The above-proposed hypothesis matters to the considered mass extinctions. One would wonder whether all interruptions or changes of long- or short-term eustatic trends triggered biotic crises. Definitely, this was not so. For instance, a trend change can be observed at the Carboniferous–Permian transition (Figure 1), but the relevant transformative event is not associated with any mass extinction. Some interruptions of a trend toward sea-level rise took place in the second half of the Jurassic, but also without biotic crises. It should be stressed that interruptions and changes of eustatic trends were, hypothetically, responsible for the vulnerable state of marine biota. In the absence of extrinsic or intrinsic perturbations, this vulnerability may not lead to mass extinctions. In other words, interruptions and changes of eustatic trends did not necessarily trigger biotic crises in the marine realm, but only increased risks of negative scenarios.

## 5. Conclusions

The use of the new eustatic reconstructions by Haq [32,33,34] and Haq and Schutter [35] for the understanding of the global sea-level changes at the intervals of the fifteen Paleozoic–Mesozoic mass extinctions permits making two general conclusions. First, a direct relationship between the considered biotic catastrophes in the marine realm and the eustatic rises/falls is absent. Second, many mass extinctions correspond to interruptions and changes of the “short-term” and “long-term” eustatic trends. Hypothetically, trend-affecting sea-level changes could facilitate or even trigger mass extinctions. The explanatory mechanisms need to be well characterized, and this is a task for further investigations. In other words, the outcomes of this study leave space for the eustatic explanation of biotic crises in the marine realm, but indicate the urgency of changing the angle of looking at this factor.

Methodologically, the present paper demonstrates that the application of episodic event analysis [38] provides a new vision of the eustatic factor of marine biota perturbations. Future research may permit making the graphical analysis of trends more accurate (for instance, the trends can be calculated as mathematical functions), and even classification of eustatic events with the power of artificial intelligence may become possible. However, the solution of such an ambitious task requires further refinement of the available eustatic curves. Moreover, better understanding of biotic crises, their magnitude, and precise timing is desirable. For instance, the only very recent discovery of the Carnian catastrophe [53] implies how incomplete the knowledge of mass extinctions can be even for such well-studied geologic time intervals as the Mesozoic.

## Figures and Tables

**Table 1:** The Paleozoic–Mesozoic mass extinctions considered in the present study.

Label *	Age **	Status	Key Literature ***
a	mid-Cambrian (end-Series 2)	minor	[40]
B	end-Ordovician (Hirnantian)	“Big Five”	[41,42]
c	Llandovery/Wenlock	minor	[43]
D	Late Devonian (Frasnian/Famennian)	“Big Five”	[44]
e	Devonian/Carboniferous	minor	[44]
f	mid-Carboniferous (late Serpukhovian)	minor	[45,46]
g	end-Guadalupian	potentially major	[47,48]
H	end-Permian	“Big Five”	[49,50,51]
i	mid-Triassic-1 (Ladinian)	possible	[52]
j	mid-Triassic-2 (Carnian)	minor	[53]
K	end-Triassic	“Big Five”	[54,55,56]
l	Early Jurassic (early Toarcian)	minor	[57,58,59]
m	Jurassic/Cretaceous	minor	[60,61]
n	Late Cretaceous (late Cenomanian)	minor	[62,63,64]
O	end-Cretaceous	“Big Five”	[65]

Notes: * the labels correspond to Figure 1 (“Big Five” mass extinctions are capitalized); ** the geologic time scale developed recently by the International Commission on Stratigraphy is followed (numerical age is not provided to avoid apparent inconsistencies between the stratigraphic scales and the available mass extinction dating); *** due to voluminous literature on some mass extinctions, a few of the most important, recent, and timing-related sources are selected, and the present paper does not attempt to summarize the literature evidence of each mass extinction (nonetheless, additional sources are cited in the text below).

**Table 2:** Eustatic context of the considered mass extinctions.

Labels *	Global Sea-Level Changes	
	Hallam and Wignall [17]	This Study
a	not recorded	strong fall and strong rise
B	fall and rise	strong falls with strong rise in between
c	-	strong fall
D	rise	weak fluctuations and strong fall
e	rise	strong fall
f	-	weak fluctuations
g	fall	strong fall
H	rise	weak fluctuations
i	-	strong rise and weak fluctuations
j	-	strong fall
K	fall	strong fall, strong rise, and weak fluctuations
l	rise	strong rise and strong fall
m	-	moderate fluctuations
n	rise	weak fluctuations and strong rise
O	rise	weak fall

Note: * see Figure 1 and Table 1.

**Table 3:** Summary of the eustatic event interpretations linked to the considered mass extinctions.

Labels *	Anomalous	“Short-Term” Record	“Long-Term” Record
a	No	ordinary	ordinary
B	Yes	transformative	interruptive
c	Yes	decreasing	quasi-interruptive
D	Yes	interruptive	interruptive
e	Yes/No	interruptive	interruptive
f	No	stabilizing	interruptive
g	Yes	stabilizing	stabilizing
H	No	facilitative	ordinary
i	No	ordinary	ordinary
j	No	ordinary	ordinary
K	Yes	interruptive	interruptive
l	Yes	transformative	decreasing
m	No	interruptive	interruptive
n	No	transformative	transformative
O	No	ordinary	ordinary

Note: * see Figure 1 and Table 1.

## Supplementary Materials

The following are available online at https://www.mdpi.com/2075-1729/10/11/281/s1, Figure S1: The Paleozoic-Mesozoic eustatic fluctuations, their trends, and mass extinctions (see text for more information).
