# Supplementary material for: Paleozoic–Mesozoic Eustatic Changes and Mass Extinctions: New Insights from Event Interpretation
Source: Life (Basel). 2020 Nov 14;10(11):281. doi: 10.3390/life10110281 (PMC7698083; doi:10.3390/life10110281)
Supplement: Supplementary file 1 [file life-10-00281-s001.pdf]

life

Supplemental Material

Paleozoic–Mesozoic Eustatic Changes and Mass Extinctions: New Insights from Event Interpretation

Dmitry A. Ruban

K.G. Razumovsky Moscow State University of Technologies and Management (the First Cossack University), Zemlyanoy Val Street 73, Moscow 109004, Russia; ruban-d@mail.ru

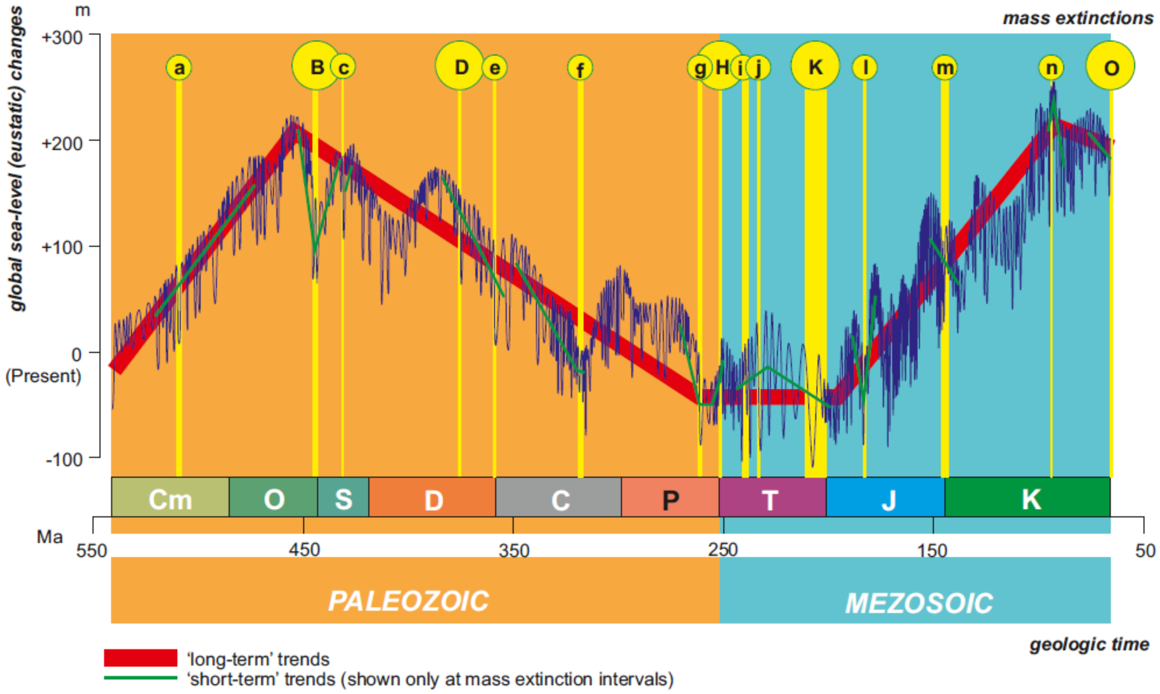

Figure S1. The Paleozoic-Mesozoic eustatic fluctuations, their trends, and mass extinctions (see text for more information).

Publisher's Note: MDPI stays neutral with regard to jurisdictional claims in published maps and institutional affiliations.

© 2020 by the authors. Submitted for possible open access publication under the terms and conditions of the Creative Commons Attribution (CC BY) license (http://creativecommons.org/licenses/by/4.0/).
